# Dimethyl diallyl ammonium chloride and diallylamin Co-polymer modified bio-film derived from palm dates for the adsorption of dyes

**DOI:** 10.1038/s41598-017-14327-7

**Published:** 2017-10-31

**Authors:** Mahjoub Jabli, Tawfik A. Saleh, Nouha Sebeia, Najeh Tka, Ramzi Khiari

**Affiliations:** 1 Chemistry Department, King Fahd University of Petroleum & Minerals, Dhahran, 31261 Saudi Arabia; 2 Textile Materials and Research, National School of Engineering (ENIM), Monastir, 5000 Tunisia; 3 Laboratory of Organic Assymetric and Homogenous Catalysis (FSM), Monastir, Tunisia; 4 High Institute of Technological Studies of Ksar Hellal, 4018 Monastir, Tunisia

## Abstract

For the first time, co-polymer of dimethyl diallyl ammonium chloride and diallylamin (PDDACD) was used to modify the films derived from the waste of palm date fruits, which were then investigated by the purification of colored aqueous solutions. The physico-chemical characteristics were identified using data color, FT-IR spectroscopy, and SEM features. The modified films were evaluated as adsorbents of Methylene Blue (MB), Direct Yellow 50 (DY50), Reactive Blue 198 (RB198) and Naphtol Blue Black (NBB). High retention capacities were achieved in the following order: The equilibrium da DY50 (14 mg g^−1^) < RB198 (16 mg g^−1^) < NBB (63.9 mg g^−1^) < MB (150 mg g^−1^). The kinetic modeling of the data revealed that the adsorption data follows the pseudo second order model. It was fitted to the Langmuir, Freundlich, Temkin, and Dubinin-Redushkevich equations, and the data best fit the Freundlich model indicating that the adsorption might occur in the heterogeneous adsorption sites. These results reveal that PDDACD modified films are valuable materials for the treatment of industrial wastewater. Moreover, the as-prepared adsorbent is economically viable and easily controllable for pollutant adsorption.

## Introduction

Water contamination could lead to several harmful effects, including the destruction of aquatic life, and even have a hazardous influence on humans. As a result, the removal of pollutants is important for controlling water quality. The treatment of water by adsorption using adsorbent is one of the most promising methods. The capacity for the pollutants’ removal is a function not only of the pore size and structure of the adsorbent but also depends on the molecular size and chemical nature of the solutes^1^. For a long time, one emergent concern has been devoted to using available low-cost materials for the removal of pollutants^2,3^. In this framework, previous studies bear with the development of many methods to treat polluted and colored water through sorption or degradation process^4–10^.

Recently, we demonstrated that 4-methyl-2-(naphthalen-2-yl)-N-propylpentanamide functionalized ethoxy-silica could be used as an efficient adsorbent to remove a wide range of dyes (acid, direct, reactive and basic) from an aqueous suspension^11^. In particular, the most frequent examples given were forest products that were proven to be competent and were used as adsorbents are palm ash^12^, rice husk^13^, sawdust and almond Shell^14,15^. Many parts of the palm plant have been extensively studied in wastewater treatment mainly trees^16^, leaf^17^, and fruits^18^. For example, the studies reported by Banat *et al*.^19^ have detailed the adsorption kinetics and isotherms of methylene blue (MB) on raw and thermally activated date pits as agricultural solid waste. Walker *et al*. have concentrated on the adsorption mechanism of the removal of heavy metals and dyes from aqueous solutions using date pits as a solid adsorbent^20^. However, to our best knowledge, no study about the valorization of the film arising from the date waste was undertaken, even in its raw or modified form, for environmental or other applications.

Problems with water are expected to grow worse in the coming decades, with water scarcity occurring globally. Addressing these problems calls for new cost-effective materials and to identify robust methods for purifying water at lower cost, while at the same time minimizing the use of chemicals and the impact on the environment.

In this work, we report the co-polymer of dimethyl diallyl ammonium chloride and diallylamin modified films derived from waste palm date fruits to obtain hybrid material as an effective, stable, and efficient adsorbent for the removal of dyes. A rapid uptake rate and high adsorption capacity were observed. The results obtained in this study deliver fundamental knowledge as well as valuable experience, which will serve as a reference for the planning and design of the polymer modification of films for removing pollutants in wastewater, where chemical production, mining sites, and mine wastes are important.

## Experimental Procedure

### Chemicals and reagents

The liquid co-polymer of dimethyl diallyl ammonium chloride and diallylamin (PDDACD) was laboratory grade. MB, RB198, DY50, and NBB were supplied from Sigma Aldrich. Their chemical structures and physical characteristics were given in Table 1. All chemical reagents were, also, purchased from Sigma Aldrich and used as laboratory grade.

**Table 1:** Chemical structures of the studied dyes and their physical characteristics: (**a**) RB198, (**b**) DY50, (**c**) NBB and (**d**) MB.

	
Reactive Blue 198 (RB198) λ_max_ = 595 nm Molecular weight (g/mol) = 882.19	Direct Yellow 50 (DY50) λ_max_ = 390 nm Molecular weight (g/mol) = 952.81
	
Naphtol blue Black (NBB) λ_max_ = 610 nm Molecular weight (g/mol) = 616.49	Methylene Blue (MB) λ_max_ = 664 nm Molecular weight (g/mol) = 319.85

### Extraction and functionalization

**Figure 1:**
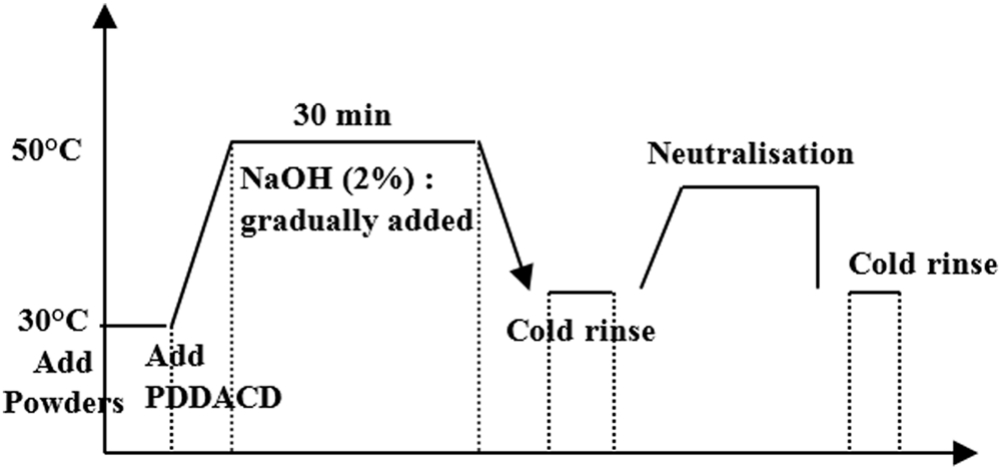
Cationization process using PDDACD.

**Figure 2:**
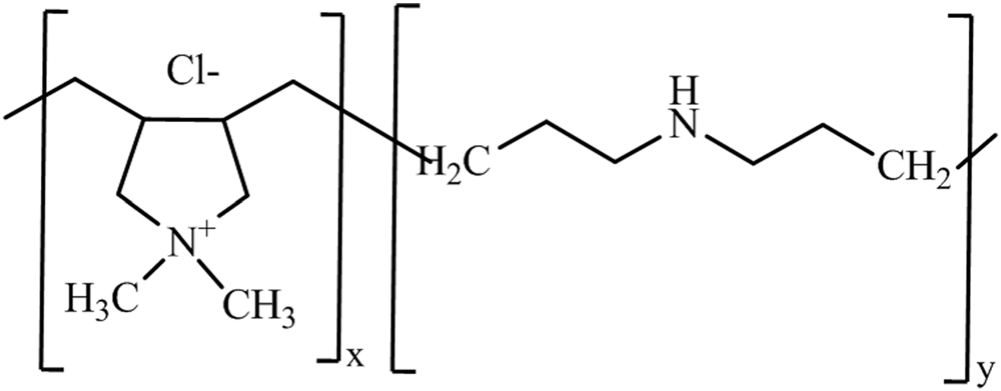
Structure of poly-dimethy-diallyl-ammonium-chloride-diallylamin-co-polymer (PD*D*ACD).

**Figure 3:**
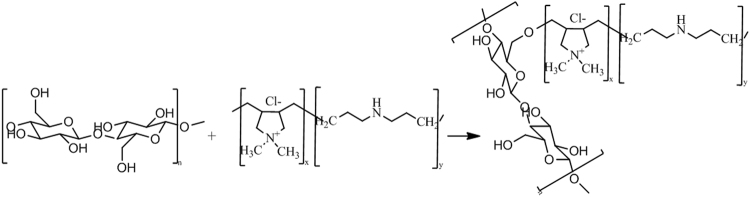
A proposed mechanism of interaction between cellulose chains of raw film and PDDACD.

The films were collected from palm dates grown from the “*Canticha”* and “*Deghla”* varieties. They were rinsed with water many times to remove the impurities deposed on the surface. The modification of the films was performed at a temperature of 50 °C for 30 mins in the presence of NaOH (2%) within a range of 0.05–5% PDDACD according to the process described in Fig. 1. PDDACD, with the structure drawn in Fig. 1, was added to the films and well mixed. Then, 2% NaOH solution was dropwise added and the mixture was stirred for 30 mins, followed by cooling. Then, the mixture was neutralized by acetic acid which was followed by washing with distilled water. Figure 2 depicts the structure of poly-dimethy-diallyl-ammonium-chloride-diallylamin-co-polymer (PDDACD). Figure 3 depicts the proposed chemical structure of the obtained dimethyl diallyl ammonium chloride and diallylamin co-polymer modified bio-film.

The obtained raw materials were ground and 40–60 mesh fractions were selected according to the standard procedures T264 cm-07 to determine their chemical composition. The chemical analysis of the film obtained from date palm was performed according to the standard methods. The evaluation of the extractive substances was carried out in various liquids according to common standards. The solubility in hot and icy water, 1% NaOH and in ethanol–toluene was established according to the TAPPI standards methods: T207 cm-08, T212 om-07, and T204 cm-07 respectively. The ash amount was established according to standard T211 om-07. The quantities of lignin, holocellulose and α-cellulose, were also evaluated using the following respective TAPPI standard methods: T222 om-06, the method of Wise *et al*.^21^ and T203 cm-99. All the experiments were duplicated and the difference between the values was within an experimental error of 5%.

### Characterization

An FT-IR apparatus (PerkinElmer 100 spectrometer, USA) was used to determine the different function groups present in the structure of film palm date waste after chemical modification. In order to obtain a good resolution of the spectrum, the spectra of the samples were obtained after 32 scans from 400 to 4000 cm^−1^ with a resolution of 4 cm^−1^.

The morphological features of the products before and after modification were analyzed using the SEM apparatus (Hitachi S-2360N). Samples were previously coated with gold using a vacuum sputter-coater in order to improve their conductivity and the quality of the SEM images. The accelerating voltage was equal to 20 kv. A spectrophotometer was employed to measure the absorbance value of dyes before and after adsorption. The whiteness index and the color coordinates of raw films were assessed using a data color instrument.

### Adsorption experiments

**Figure 4:**
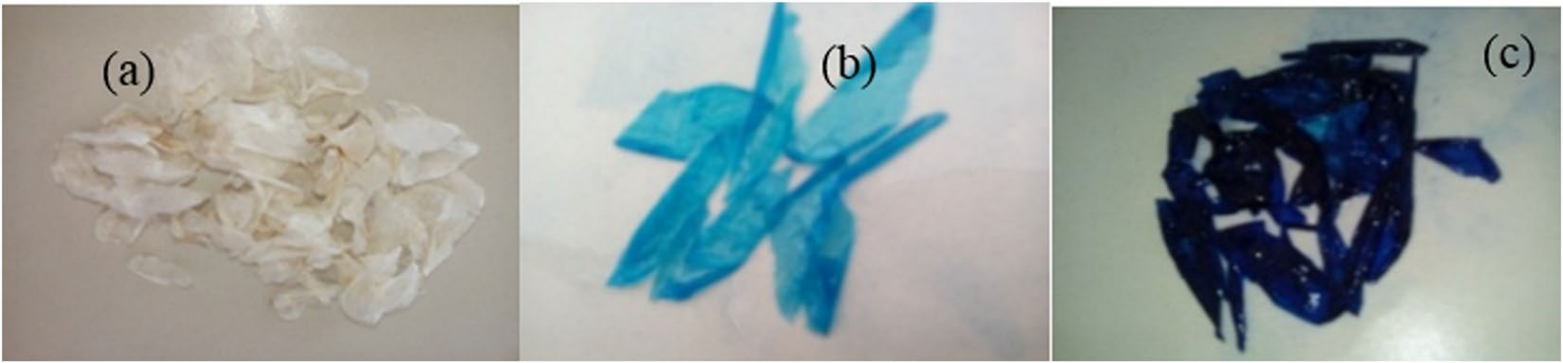
Photos of (**a**) residual films (**b**) after contact with MB [10 mg/L] and (**c**) [30 mg/L].

Kinetic studies were carried out by agitating a series of flasks containing 50 mL of dye solutions of an initial concentration of 30 mg.L^−1^ with 0.05 g amount of adsorbent (unmodified and modified film waste) with a constant agitation speed (50 rpm). Agitation was provided for 4 h after which equilibrium was reached, Fig. 4. After reaching an equilibrium state, the contents were filtered to separate the adsorbents from the suspension using a sintered glass. The p*H* values were ranged from 3 to 9 at temperatures from 25 to 60 °C to examine the effect of the experimental conditions on the adsorption phenomenon. All the experiments were duplicated and the difference between the values was within the experimental error of 5%.

## Results and Discussion

### Chemical analysis

The average results of the chemical analysis of the bio-films obtained from date palm were determined (Table 2). It was registered that the starting raw material was characterized by relatively high amounts of 12.3% in hot water and 6.09% in cold water extractives. Extractions were carried out under alkaline conditions which yielded a very high content, i.e. 20%, which probably indicates oligosaccharide and lignin-rich materials^22,23^. The ash content is equal to 3.46%. Whereas the ethanol-toluene extractives (5.84%), holocellulose (63.55%) and α-cellulose contents (45.41%) are comparable to those of other annual plants or agricultural crops^24^. Klason lignin was found to be relatively lower (14.23%). The polysaccharide content is close to that associated with wood materials, which makes the waste a very promising candidate to investigate for the isolation of cellulose as composite materials and/or papermaking, and/or as a substrate for cellulose derivatives.

**Table 2:** The chemical composition of the obtained bio-films from the date palm.

	R (%)	Standard method
Cold water extractives	6.09	T207 cm-08
Hot water extractives	12.30	T207 cm-08
1% NaOH extractives	19.29	T212 om-07
Solubility in ethanol–toluene	5.84	T204 cm-07
Ash	3.46	T211 om-07
Lignin	14.23	T222 cm-99
Holocellulose	63.55	Wise *et al*., 1946
Hemicellulose	18.14	**
α-cellulose	45.41	T203 cm-99

**The hemicellulose content was calculated by subtracting the cellulose content from the holocellulose content.

### Physical characteristics

**Figure 5:**
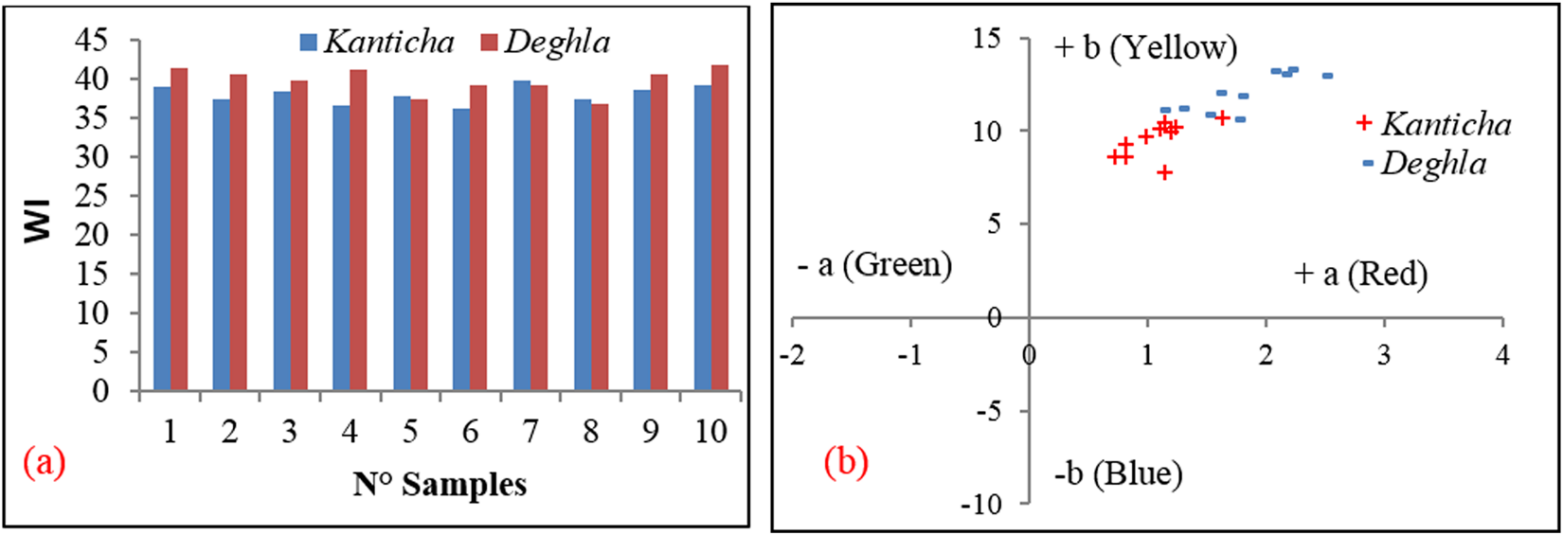
Variation of: (**a**) WI for films from the Kanticha and *Deghla* varieties and (**b**) evolution of their yellowish and reddish color.

The major produced varieties of palm date in Tunisia are namely: Deghla, Kenticha, Alig, KentaGenda and Kosbi differentiated by the aspect of their fruit. Herein, the WI variation of the raw films for two abundant varieties, Kanticha and deghla, in our region (Sidi Bouzid, Tunisia) was measured in order to examine variations in the native color. From Fig. 5a, only a slight change in the WI value inside the whole variety is observed. In fact, the WI values varied from 36.2 to 39 and from 36.7 to 42 for Kanticha and Deghla, respectively. Applying the equation 1, the Cv (%) allows us to comment on the homogeneity of the serial of each variety. This value was found to be equal to 2.06% and 1.65% for the Kanticha and Deghla varieties, respectively.

1 $$CV\,( \% )=\frac{\sqrt{\frac{1}{{\rm{n}}}(Xi-M)2}}{{\rm{M}}}$$

Contrary to *Deghla*, the *Kanticha* variety bears a dry fruit in nature and consequently, the bio-films could be easily separated from the date waste. As a result, it was selected as a candidate for further dye sorption experiments.

The color parameters (L*, a* and b*) were, also, assessed and the results were tabulated in Table 3. The L* parameter is associated with the lightness of the color and moves from the top (value: 100, white) to bottom (value: 0, black). For the studied films, it varies between 69.71 and 64.69 CIELAB units indicating a perceptible brightness in the studied films. The value of a* [associated with greenness (−) to redness (+) changes] varied from 0.72 to 1.63, conferring the products with a reddish color. The changes in b* [associated with blueness (−) to yellowness (+) changes] ranges from 7.79 to 10.75, conferring the products with a yellowish color. The comparison between the *Kanticha* and *Deghla* varieties reveals that the latter is more red and yellow in color.

**Table 3:** Color coordinates for *Kanticha* and *Deghla* varieties.

N°	*Kanticha variety*			*Deghla variety*		
	L*	a*	b*	L*	a*	b*
1	64.69	1.14	7.79	70.27	1.12	11.16
2	67.58	0.81	8.61	68.85	1.59	12.03
3	67.56	1.24	10.18	69.88	2.05	13.23
4	66.72	1.63	10.75	70.49	1.5	10.91
5	68.27	1.14	10.43	68.89	1.27	11.25
6	68.94	0.99	9.71	69.9	2.2	13.28
7	68.71	0.82	9.27	67.08	2.14	13.05
8	68.49	1.11	10.09	70.73	1.78	11.9
9	69.47	0.72	8.65	69.26	1.75	10.66
10	69.71	1.19	9.99	67.63	2.48	13

### FT-IR investigations

**Figure 6:**
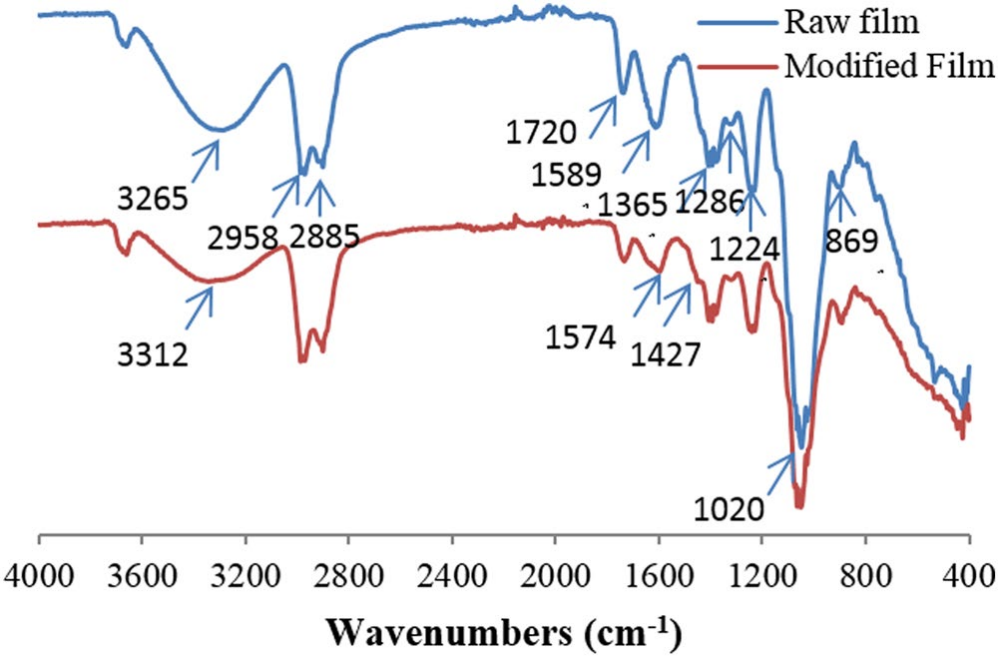
FT-IR spectra of raw and modified film.

The FT-IR spectra of raw and functionalized films are given in Fig. 6. The results depicted that the raw film showed a strong broad band at around 3265 cm^−1^ corresponding to the OH stretching mode. The cellulose structure of the film is confirmed by the presence of many characteristic bands: the peak at 869 cm^−1^ (an amorphous region in cellulose) is assigned to *ß*-glucosidic linkages^25^. The band at 1020 cm^−1^ is attributed to the C-OH stretching vibration of the cellulose backbone (*ʋ*C-O secondary alcool)^26^. The symmetric CH bending of the methoxyl groups was observed at 1365 cm^−1^ ^27^. The two bands at around 2885 and 2958 cm^−1^, are related to -OCH_3_ methoxyl C-H stretching and C-H stretching groups^28,29^. The peaks present at 1720 (C=O linkage) and 1224 cm^−1^ (aromatic skeletal vibration) are characteristic groups of lignin and hemicelluloses^30^. The bands at 1589 and 1286 cm^−1^ are assigned to C=C and C–O stretching vibrations of different groups present in lignin^28^.

Compared with the IR spectrum of raw film, the spectrum of the modified film exhibits the shifting of the band at 3265 cm^−1^ (OH stretching groups) to 3312 cm^−1^. This confirms the addition of ammonium ions arising from the PDDACD structure. It can be also observed that there is an appearance of two new peaks at 1427 and 1574 cm^−1^ which are attributed to the C-N stretching vibration and N–H in the secondary amine (-NH), respectively^31^. These results corresponded to the quaternary ammonium salt groups which reacted on the cellulose backbone.

### SEM characterization

**Figure 7:**
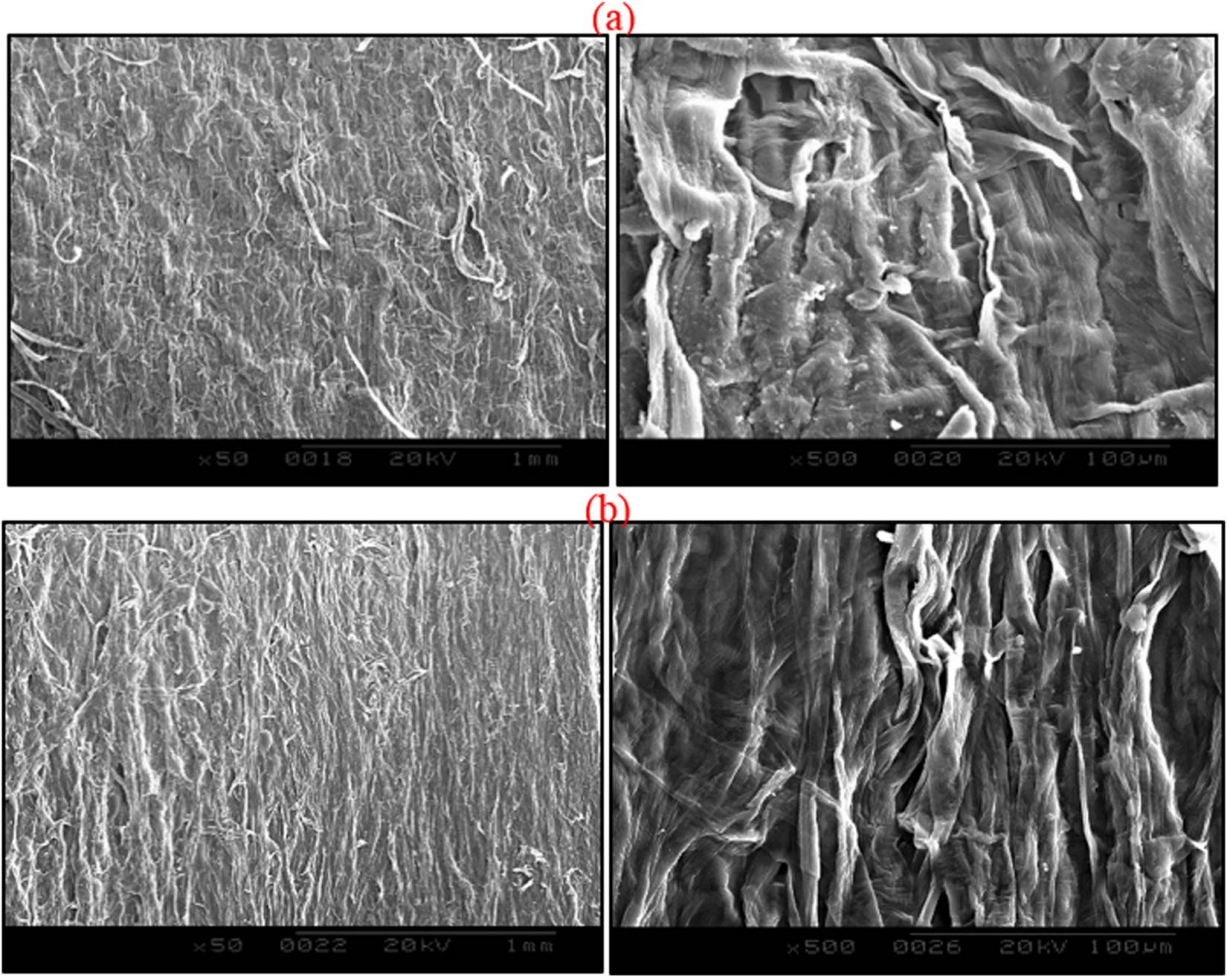
Micrograph of (**a**) unmodified and (**b**) modified films (×50 and 500).

The morphological features of the films were examined by Scanning Electron Microscopy and the results were given in Fig. 7. The SEM image (Fig. 7a) shows a micrograph of the unmodified films waste surface where a system of shallow parallel grooves is observed. Figure 7b depicts the micrographs of the film-PDDACD surface. It can be concluded, based on these micrographs, that there are no clear changes in surface morphology and the cationized surfaces are slightly smoother than that of the unmodified films. It can be concluded that the cationization does not alter the fiber’s physical structure. This is an advantage of the treatment compared to some other polymer material treatments (i.e. chitosan) which produce a degree of stiffness.

### Adsorption of dyes on unmodified and modified films

The interaction of film chains with the studied representative adsorbates is conditioned, not only by the presence of functional groups on the surface of either the adsorbent or the adsorbate but also by several experimental parameters including p*H* value, duration of contact, initial adsorbate concentration, temperature range, etc.

### Effect of pH value

**Figure 8:**
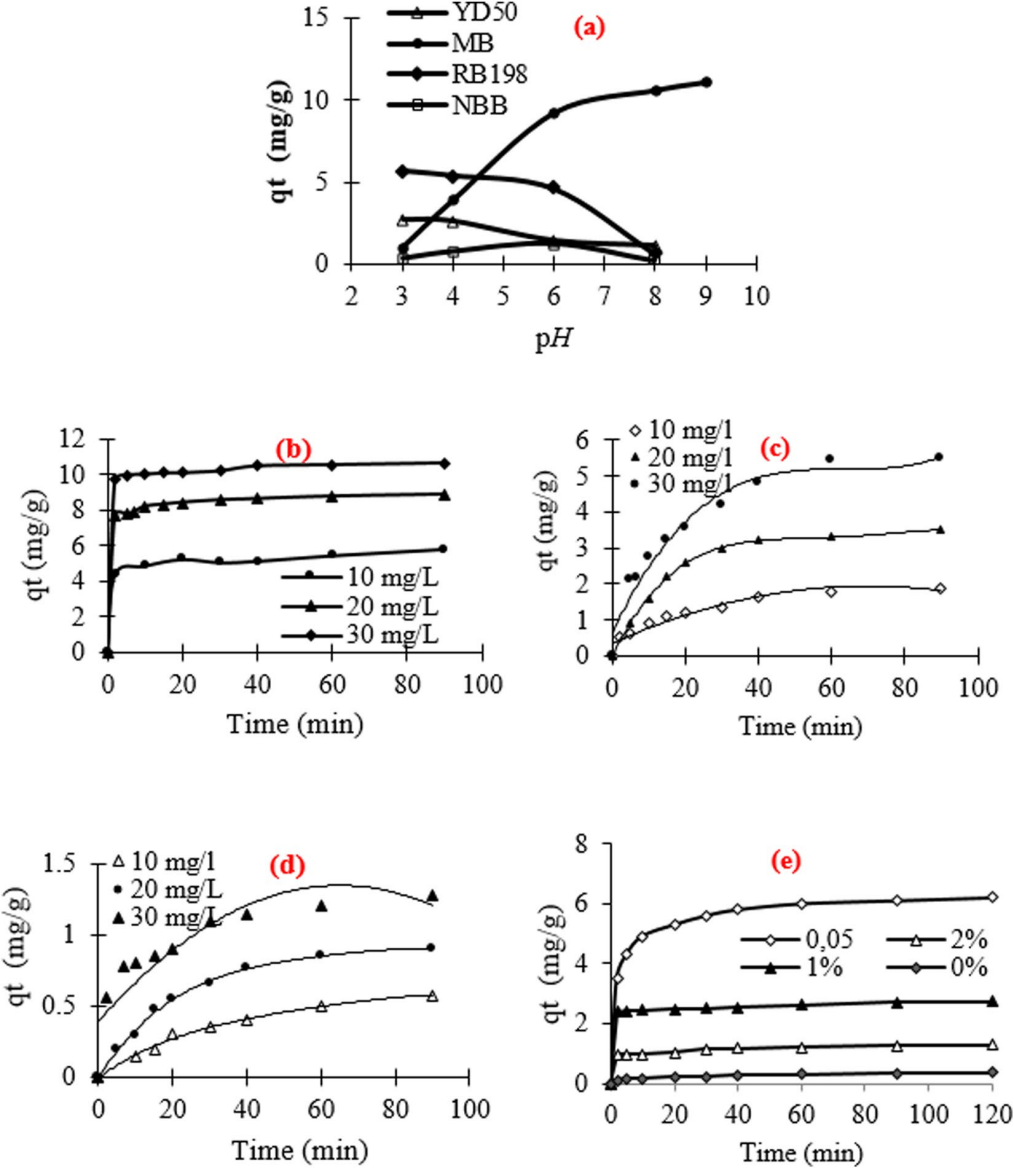
(**a**) Effect of pH on the adsorption of dyes (c_0_ =  30 mg/L, t = 1 h, T = 25 °C), Change of q_t_ against time for the adsorption of: (**b**) MB, (**c**) RB198, (**d**) DY50 and (**e**) NBB (T = 25 °C, pH = 6, c_0_ = 30 mg/L) on the surface of bio-films.

The effect of p*H* on the adsorption of DY50, MB, RB198, and NBB is given in Fig. 8a. The adsorption capacity achieved its maximum at pH 4 for RB198 and DY50, p*H* 8 for BM and p*H* 6 for NBB. For example, for MB, the adsorption capacity increases from 1 to 10.5 mg.g^−1^ when the pH ranges from 3 to 9. These lower q_t_ values observed for MB, at an acidic pH, might be due to the presence of excess H^+^ ions competing with dye cations for the available adsorption sites^32^. The results agree with other reports studying the removal of MB on aluminum industry waste and on low-cost activated carbon derived from agricultural waste material, respectively^2,33^.

### Effect of time

The variation of the adsorbed quantity of the film waste versus time is given in Fig. 8b–e. As globally observed, the sorption equilibrium was rapidly achieved for dye concentrations ranging from 10 to 30 mg g^−1^. Indeed, only 5 minutes of contact adsorbent-MB were sufficient to achieve equilibrium, 40 minutes for RB198 and 80 minutes for DY50. This difference in the rate and capacity of sorption is explained based on the reactivity of the film wastes, the molecular weight and the nature of the dye itself (Table 1). In addition, the functionalization with PDDACD was found to enhance significantly the adsorption of NBB. The adsorbed amount is about 5.9 mg.g^−1^(C_0_ = 30 mg.L^−1^) for the optimum dose of PDDACD (0.05%). However, it does not exceed 0.25 mg.g^−1^ for the raw film under the same conditions. In addition, it can also be noticed that the adsorbed amount of NBB decreases with the increase in the cationic agent dose. For example, q_t_ decreases from 5.9 mg g^−1^ (optimum dose of PDDACD) to 1.29 mg g^−1^ for the same experimental conditions using a high cationic dose equal to 2%. The trend in capacity removal q_t_ can be explained by the effect of an ionic attraction between the PDDACD cationic groups and the dye anionic groups. Nevertheless, for modified films, q_t_ is slightly reduced at high doses of the cationic agent. These trends are due to the formation of the dye-PDDACD complex on the film waste surface which, because of steric hindrance, blocks dye diffusion through functional hydroxyl groups into the cellulosic film. Similar behaviors were also observed in the reports by Mouxiou *et al*.^34^ when studying the cationic surfactants and their interaction with reactive dyes and during the investigation of Nebojša Ristić *et al*.^35^ which describes the cationic modification of cotton fabrics and reactive dyeing characteristics.

### Effect of temperature

**Figure 9:**
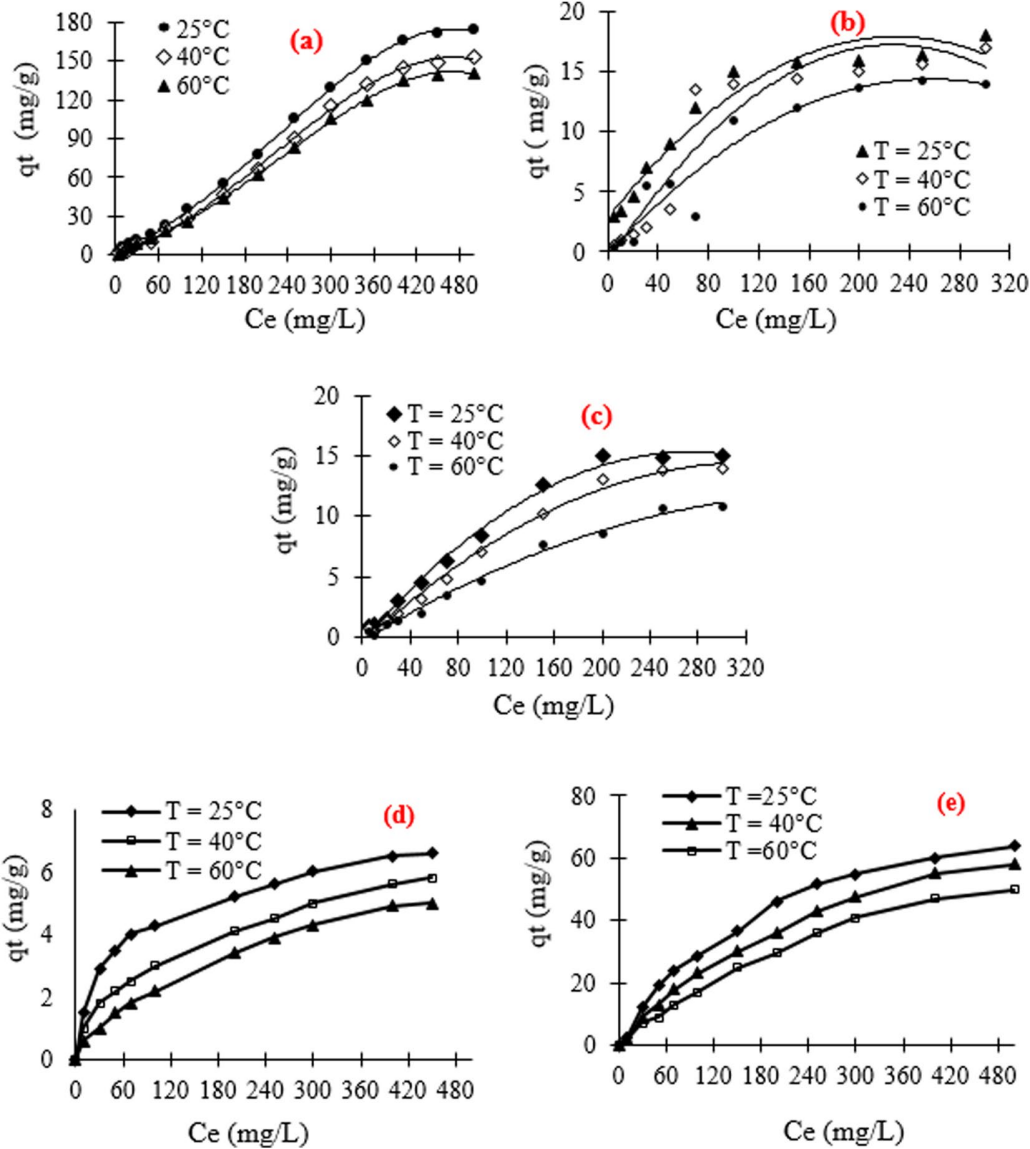
Evolution of q_t_ versus ce: (**a**) MB, (**b**) RB198, (**c**) DY50, (**d**) Variation of temperature for NBB Unmodified film and (**e**) Variation of temperature for NBB cationized film.

The evolution of the adsorbed amount of MB, RB198, DY50 and NBB (Fig. 9) on the surface of the unmodified and functionalized films is studied as a function of temperature in order to comment on the exothermicity or endothermicity of the process. As depicted for the four investigated dyes, the adsorption process using film waste as adsorbent follows an exothermic mode in the range of 25–60 °C. As an example, at room temperature, when the Ce of MB increases from 5 to 500 mg.L^−1^, the adsorbed amount of dye achieved its maximum at about 150 mg.g^−1^. The maximum adsorbed quantities, at 25 °C, are to be 16 mg.g^−1^ and 14 mg.g^−1^ for RB198 and DY50, respectively.

The increase in the Ce values exhibits that the q_t_ increases from 6.6 mg.g^−1^ for the unmodified film (Fig. 9d) waste to 63.9 mg g^−1^ after its functionalization with PDDACD. This proves the efficiency to modify the cellulosic chains using this cationic copolymer in the removal of anionic dyes. As globally observed for all dyes, the high capacity removal proves that this low-cost agricultural waste could be considered as an efficient adsorbent. These sorption amounts follow the order: DY50 (14 mg.g^−1^)  < RB198 (16 mg.g^−1^)  < NBB (63.9 mg.g^−1^) < MB (150 mg.g^−1^). This difference in capacity removal was explained by the functional groups present in the structure of each dye and their molecular weight. Indeed, DY50 and RB198 exhibited high MW values (>882 g.mol^−1^).

The registered maximum adsorption capacity for the removal of MB using raw films (150 mg.g^−1^) as the adsorbent is compared to other adsorbents gathered from the literature (Table 4). This amount of MB removal is so very interesting and thus the studied bio-films could be seen as a good adsorbent. In fact, this value is more important compared to hydroxyapatite modified with λ-Carraghenan (98.23 mg.g^−1^), Almond Shell waste (84.9 mg.g^−1^) and Elaeagnus angustifolia (75.75 mg.g^−1^). It is three times higher compared to Pyrolytic tire Char (50 mg/g). It is seven times more important than the zeolite ZK (21.41 mg.g^−1^). It is nine times important than SDBS-modified ZSM-5 (15.68 mg.g^−1^) and it is also fourteen times important than hydroxysodalite (10.82 mg.g^−1^).

**Table 4:** The maximum adsorption capacities (mg.g^−1^) of MB from the literature by other adsorbents.

Samples	q_m_ (mg.g^−1^)	References
Almond Shell waste	84.9	36
Hydroxyapatite	98.23	37
Zeolite ZK	21.41	38
SDBS-modified ZSM-5	15.68	38
Hydroxysodalite	10.82	38
Elaeagnus angustifolia	75,75	39
Pyroltic tire Char	50	40
Bio-films from palm date	150	Current study

### Kinetic modeling

The correlation of the experimental kinetic data with theoretical equations allows us to better understand the mechanism of the retention of all the studied dyes on the surface of unfunctionalized and functionalized film wastes during the sorption phenomenon. The detailed linear forms of these equations were given in the previous works^10^. In the present work, experimental data are discussed based on the coefficient regression R^2^ and the calculated SSE values (Table 5). As globally observed, for all the studied dyes using unfunctionalized (Figure S1) and functionalized film (Figure S2) waste as adsorbents, the values of R^2^ are found to be more important, along with the pseudo second order equation (R^2^ > 0.85) compared to the pseudo-first-order. This was also confirmed by the fact that the calculated q_e_ values for the pseudo-second-order kinetic model show good agreement with the experimental q_e_ values (0.0005 < SSE < 0.044).

**Table 5:** Summarized kinetic constants for the adsorption of different dyes on the surface of unmodified and cationized films.

C_0_ (mg/L)	Pseudo-first-order				Pseudo-second-order				Elovich			Diffusion	
	K_1_	q_e_	R^2^	SSE	K_2_	q_e_	R^2^	SSE	α	β	R^2^	K_1_	R^2^
**MB**													
10	0.0089	2.135	0.66	0.461	0.093	5.76	0.99	0.009	6.654	0.657	0.623	0.4059	0.511
20	0.01	1.891	0.55	0.87	0.003	8.888	0.99	0.002	28.58	0.754	0.529	0. 586	0.412
30	0.0098	1.829	0.48	1.1	0.153	10.66	0.99	0.002	303.6	1.119	0.475	0.6697	0.366
**RB198**													
10	0.007	1.824	0.9	0.006	0.054	2.014	0.97	0.017	0.468	2.493	0.975	0.1988	0.952
20	0.0098	2.9	0.86	0.075	0.099	3.852	0.97	0.044	0.778	1.207	0.971	0. 4009	0.904
30	0.0119	4.301	0.92	0.146	0.021	5.875	0.97	0.05	1.656	0.883	0.944	0.564	0.93
**DY50**													
10	0.0028	1.213	0.94	0.079	0.049	0.724	0.85	0.018	0.068	7.553	0.913	0.0657	0.98
20	0.0038	1.39	0.85	0.06	0.058	1.055	0.94	0.019	0.143	4.57	0.957	0.1042	0.95
30	0.0038	1.376	0.71	0.012	0.14	1.32	0.99	0.0005	0.453	3.731	0.937	0.12	0.83
**NBB**													
0%	0.003	0.494	0.838	0.015	0.356	0.378	0.983	0.002	0.11	13.568	0.97	0.036	0.926
0.05%	0.009	2.452	0.801	0.416	0.078	6.305	0.999	0.011	5.2	0.8579	0.82	0.099	0.585
1%	0.005	0.82	0.472	0.212	0.342	2.74	0.999	0.001	9.09	2.504	0.52	0.179	0.416
2%	0.004	0.494	0.621	0.088	0.373	1.309	0.998	0.002	1.84	4.697	0.69	0.529	0.672

### Analysis of isotherms via Langmuir, Freundlich and Temkin and Dubinin

The adsorption isotherms allow us to predict the feasibility of the adsorption phenomenon and represent the mechanism for the interaction between the adsorbent and the adsorbates at the studied temperatures.

Table 6 gives the different parameters gathered from the linearized data throughout Langmuir, Freundlich, Temkin, and Dubinin. The relatively low correlation coefficients show that the Langmuir isotherm (Figure S3) has a poor agreement with the experimental data which suggests that the adsorption phenomenon does not occur on a single surface. On the contrary, the Freundlich isotherm fits the experimental data quite well with high correlation coefficients (R^2^ > 0.78) for the four studied dyes. The consistency of the Freundlich isotherm with the data reveals that the adsorption might occur in the heterogeneous adsorption sites. However, the adsorption of NBB (Figure S4) on raw films could be described by the Langmuir equation. As globally observed, the studied fibers are moderate adsorbents (1 < n < 2) for the four studied dyes but poor sorbents for NBB (n < 1) in the case of the unfunctionalized film at temperatures higher than the ambient^8,41–45^.

**Table 6:** Summarized constants values of Langmuir, Freundlich, Temkin, and Redushkevich for the adsorption of the different dyes on the surface of raw and cationized films.

T (°C)	Langmuir			Freundlich			Temkin			Dubinin				
	q_m_	R^2^	SSE	K_F_	n	R^2^	B	A_t_	R^2^	qm	E	R^2^	SSE	
**RB198**														
25	16.52	0.81	0.41	0.392	1.505	0.781	2.955	0.2	0.946	7.0766	40.8	0.84	0.447	
40	17.85	0.81	0.67	0.177	1.292	0.924	2.834	0.13	0.9609	5.2221	19.6	0.718	0.47	
60	20.242	0.696	1.022	0.1	1.202	0.97	2.443	0.1	0.9092	3.632	28.9	0.63	0.488	
**MB**														
25	48.309	0.728	8.379	0.022	1.148	0.985	4.811	0.12	0.89	5.8814	18.9	0.535	11.2	
40	51.282	0.78	6.781	0.009	1.113	0.995	4.19	0.11	0.896	6.0122	21.3	0.641	9.79	
60	25.188	0.917	7.72	0.004	1.141	0.992	2.564	0.12	0.9	4.1768	35.4	0.739	9.12	
**DY50**														
25	48.309	0.728	2.02	0.193	1.148	0.985	4.811	0.12	0.89	5.8814	18.9	0.535	0.8	
40	39.215	0.637	1.614	0.136	1.13	0.977	3.752	0.13	0.88	5.6933	28.9	0.657	0.7	
60	23.809	0.739	0.92	0.091	1.139	0.983	2.329	0.15	0.8684	3.8098	50	0.737	0.41	
**NBB: Unmodified film**														
25	7.132	0.057	0.997	0.053	1.243	2.939	0.89	1.359	0.929	0.98	5.092	25	0.876	0.15
40	6.707	0.029	0.99	0.09	0.595	2.211	0.94	1.3	0.348	0.98	4.211	28.86	0.805	0.158
60	6.215	0.02	0.979	0.12	0.319	1.833	0.94	1.247	0.256	0.97	3.364	40.824	0.741	0.16
**NBB: Modified film**														
25	84.74	0.014	0.87	2.085	2.067	1.44	0.84	15.52	0.238	0.98	38.08	17.149	0.927	2.58
40	86.2	0.009	0.77	2.82	1.478	1.401	0.87	14.78	0.171	0.98	35	21.32	0.895	2.29
60	75.18	0.008	0.79	2.52	1.266	1.432	0.89	12.23	0.172	0.95	26.46	35.355	0.704	2.35
**Thermodynamic parameters**														
					∆H* (KJ.mol ^**−1**^ **)**			∆S*(J.mol ^**−1**^ **)**			∆G* (KJ.mol ^**−1**^ **)**			
			**T (°C)**		**25**	**40**	**60**	**25**	**40**	**60**	**25**	**40**	**60**	
Unmodified film			RB198		−28.255			−132			11.294	13.285	15.939	
			DY50		−0.117			−47			14.149	15.825	15.825	
			NBB		−24.152			−105			7.273	8.855	10.964	
			MB		−0.389			−50			14.744	15.506	16.521	
Functionalized film			NBB		−12.071			76			10.804	11.956	13.491	

Thereafter, the thermodynamic parameters *ΔH*° and *ΔS*° were computed from the slope and the intercept of the linear plot of ln *Kl* vs. 1/T (Figure S5) and the results are summarized in Table 6. It can be observed that the enthalpy values are negative. This suggests that the interaction of the four studied dyes within the film is exothermic. This result agrees well with both the decrease in the capacity removal with temperature values and with the decrease of the adsorption energy constants (B) calculated from the Temkin equation. The positive values of ΔG* and negative values of ΔS* means the non-spontaneous reaction and the decrease of the disorder, respectively. However, in the case of the adsorption of NBB using functionalized films as adsorbents, the cationization allows for the increase in the disorder of the system (Table 6).

### Immobilization mechanism

**Figure 10:**
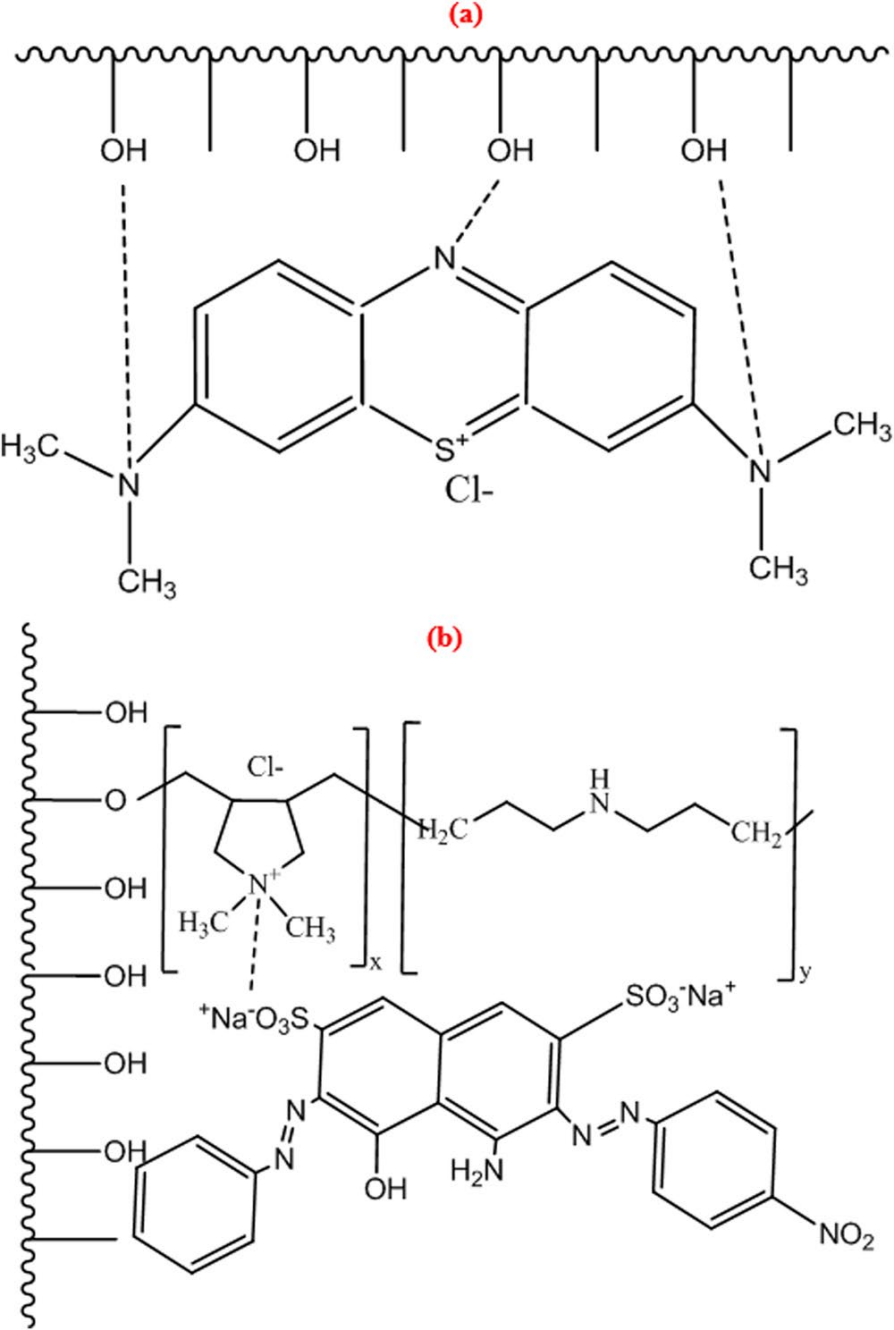
Proposed mechanism of interaction between (**a**) MB and raw film chains and (**b**) cationized film chains and NBB.

Indeed, the film chains could interact with the MB molecules via the hydrogen bonding mode through the presence of nitrogen atoms and hydroxyl groups in the structure of MB and cellulosic chains, respectively, Fig. 10(a). On the other hand, after cationisation, the ionic ammonium added on the surface of cellulosic chains might react with NBB via ionic interactions between the N^+^ and the SO_3_^−^ groups, Fig. 10(b).

## Conclusions

In summary, we have developed a new adsorbent which consists of a co-polymer of dimethyl diallyl ammonium chloride and diallylamin modified films derived from the waste of palm dates. The hybrid material showed efficient removal of dyes from polluted water. The good stability, fast adsorption rate, high adsorption capacity, and excellent pH tolerance were validated by using a batch separation mode. The capacities removal follows the order: DY50 (14 mg.g^−1^) <RB198 (16 mg.g^−1^) <NBB (63.9 mg.g^−1^) <MB (150 mg.g^−1^). The pseudo-second-order was adequate to describe the experimental data. The modeling of the isotherms reveals that the Langmuir model is more suitable to describe the adsorption data. The values of the thermodynamic parameters suggest that the phenomenon is exothermic with a non-spontaneous reaction. Moreover, the results from the chemical composition investigation exhibited that the polysaccharide content is similar to that associated with wood materials, which supports the conclusion that the films have the potential to isolate cellulose as composites, for papermaking and as a substrate for cellulose derivatives, and can thus be utilized for a wide range of applications.

## Electronic supplementary material

Supplementary file

## References

[1] Kyzas GZ, Kostoglou M (2014). Green Adsorbents for Wastewaters: A Critical Review. Materials.

[2] Gupta VK, Ali I, Saini VK (2004). Removal of rhodamine B, fast green, and methylene blue from wastewater using red mud, an aluminum industry waste. Industrial Engineering Chemical Research.

[3] Saleh, T.A. Mercury sorption by silica/carbon nanotubes and silica/activated carbon: a comparison study. *Journal of Water Supply: Research and Technology-Aqua* **64**(8), 892–903 (2015).

[4] Jabli M, Baouab MHV, Roudesli MS, Bartegi A (2011). Adsorption of Acid Dyes from Aqueous Solution on a Chitosan-cotton Composite Material Prepared by a New Pad-dry Process. Journal of Engineered Fibers and Fabrics.

[5] Jabli M, Aloui F, Ben Hassine B (2013). [Copper (II)/Cellulose-Chitosan] Microspheres Complex for Dye Immobilization: Isotherm, Kinetic and Thermodynamic Analysis. Journal of Engineeing Fibers and Fabrics.

[6] Gupta VK (2012). Photo-catalytic degradation of toxic dye amaranth on TiO2/UV in aqueous suspensions. Materials Science and Engineering C.

[7] Mittal A, Mittal J, Malviya A, Kaur D, Gupta VK (2010). Decoloration treatment of a hazardous triarylmethane dye, Light Green SF (Yellowish) by waste material adsorbents. Journal of Colloid and Interface Science;Volume.

[8] Mittal A, Kaur D, Malviya A, Gupta VK (2009). Adsorption studies on the removal of coloring agent phenol red from wastewater using waste materials as adsorbents. Journal of Colloid and Interface Science.

[9] Mittal A, Mittal J, Malviya A, Gupta VK (2009). Adsorptive removal of hazardous anionic dye “Congo red” from wastewater using waste materials and recovery by desorption. Journal of Colloid and Interface Science.

[10] Gupta VK (2012). Chemical treatment technologies for waste-water recycling—an overview. RSC Advances.

[11] Jabli M, Baccouch W, Hamdaoui M, Aloui F, Ben Hassine B (2016). Removal of a wide range of dyes using 4-methyl-2-(naphthalen-2-yl)-N-propylpentanamide-functionalized ethoxy-silica and raw silica. Journal of Textile Institute.

[12] Saleh, T. A. Advanced Nanomaterials for Water Engineering, Treatment, and Hydraulics (Advances in Environmental Engineering and Green Technologies), (first ed.), IGI Global (2017), ISBN-13: 978-1522521365.

[13] Malik PK (2003). Use of activated carbons prepared from sawdust and rice-husk for adsorption of acid dyes: a case study of Acid Yellow 36. Dyes and Pigments.

[14] Al-Amoudi O, Al-Homidy AA, Maslehuddin M, Saleh TA (2017). Method and Mechanisms of Soil Stabilization Using Electric Arc Furnace Dust. Scientific Reports.

[15] Ismal OE, Yıldırım L, Özdogan E (2014). Use of almond shell extracts plus biomordants as effective textile dye. Journal of Cleaner Production.

[16] Belala Z, Jeguirim M, Belhachemi M, Addoun F, Trouvé G (2011). Biosorption of basic dye from aqueous solutions by date Stones and Palm-Trees Waste: Kinetic, equilibrium and thermodynamic studies. Desalination.

[17] El-Shafey EI, Ali SNF, Al-Busafi S, Al-Lawati HAJ (2016). Preparation and characterization of surface functionalized activated carbons from date palm leaflets and application for methylene blue removal. Journal of Environmental Chemical Engineering.

[18] Azharul Islam MD, Tan IAW, Benhouria A, Asif M, Hameed. BH (2015). Mesoporous and adsorptive properties of palm date seed activated carbon prepared via sequential hydrothermal carbonization and sodium hydroxide activation. Chemical Engineering Journal.

[19] Banat F, Al-Asheh S, Al-Makhadmeh L (2003). Evaluation of the use of raw and activated date pits as potential adsorbents for dye containing waters. Process Biochemistry.

[20] Walker G, Ahmad MNM (2010). Adsorption mechanisms of removing heavy metals and dyes from aqueous solution using date pits solid adsorbent. Journal of Hazardous Materials.

[21] Wise LE, Murphy M, D’Addieco AA (1946). Chlorite holocellulose: its fractionation and bearing on summative wood analysis and on studies on the hemicellulose. Paper Trade Journal.

[22] Khiari R, Mhenni MF, Belgacem MN, Mauret E (2010). Chemical composition and pulping of date palm rachis and Posidonia oceanica - A comparison with other wood and non-wood fibre sources. Bioresource Technology.

[23] Mansouri S (2012). Chemical composition and pulp characterization of Tunisian vine stems. Industrial Crops and Products.

[24] Mechi N, Khiari R, Ealoui L, Belgacem MN (2016). Preparation of paper sheet from cellulosic fibres obtained from Prunus amygdalus and Tamarisk sp. Cellulose Chemistry and Technology.

[25] De Rosa IM, Kenny JM, Puglia D, Santulli C, Sarasini F (2010). Morphological, thermal and mechanical characterization of okra (Abelmoschus esculentus) fibres as potential reinforcement in polymer composites. Composite Science Technology.

[26] Bessadok A, Marais S, Roudesli S, Lixon C, Métayer M (2008). Influence of chemical modifications on water-sorption and mechanical properties of Agave fibres. Composite,Part A.

[27] Adel AM, Abd El-Wahab ZH, Ibrahim AA, Al-Shemy MT (2010). Characterization of microcrystalline cellulose prepared from lignocellulosic materials. Part I. Acid catalyzed hydrolysis. Bioresource Technology.

[28] Saleh TA, Al-Shalalfeh MM, Al-Saadi AA (2016). Graphene Dendrimer-stabilized silver nanoparticles for detection of methimazole using Surface-enhanced Raman scattering with computational assignment. Scientific reports.

[29] Saleh TA, Gupta VK (2014). Processing methods, characteristics and adsorption behavior of tire derived carbons: a review. Advances in colloid and interface science.

[30] Saleh TA, Rachman IB, Ali SA (2017). Tailoring hydrophobic branch in polyzwitterionic resin for simultaneous capturing of Hg (II) and methylene blue with response surface optimization. Scientific Reports.

[31] Roy S, Das PK (2008). Antibacterial hydrogels of amino acid-based cationic amphiphiles. Biotechnology and Bioengineering.

[32] Vadivelan V, Kumar KV (2005). Equilibrium, kinetics, mechanism, and process design for the sorption of methylene blue onto rice husk. Journal of colloid Interface Science.

[33] Singh KP, Mohan D, Sinha S, Tondon GS, Gosh D (2003). Color removal from wastewater using low-cost activated carbon derived from agricultural waste material. Industrial Engineering Chemistry Research.

[34] Mouxiou E, Eleftheriadis I, Nikolaidis N, Tsatsaroni E (2008). Reactive dyeing of cellulosic fibers: Use of cationic surfactants and their interaction with reactive dyes. Journal of Applied Polymer Science.

[35] Nebojša R, Ivanka R (2012). Cationic Modification of Cotton Fabrics and Reactive Dyeing Characteristics. Journal of Engineered Fibers and Fabrics.

[36] Jabli M, Emna G, Nouha S, Mohamed H (2017). Almond shell waste (Prunus dulcis): Functionalization with [dimethy-diallyl-ammonium-chloride-diallylamin-co-polymer] and chitosan polymer and its investigation in dye adsorption. Journal of Molecular Liquids.

[37] Hassen A, Jabli M, Hatem M (2017). Synthesis, characterization of hydroxyapatite-lambda carrageenan, and evaluation of its performance for the adsorption of methylene blue from aqueous suspension. Journal of Applied Polymer Science.

[38] EL-Mekkawi DM, Ibrahim FA, Selim MM (2016). Removal of methylene blue from water using zeolites prepared from Egyptian kaolins collected from different sources. Journal of Environmental Chemical Engineering.

[39] Rahimdokht M, Pajootan E, Arami M (2016). Central composite methodology for methylene blue removal by Elaeagnus angustifolia as a novel biosorbent. Journal of Environmental Chemical Engineering.

[40] Makrigianni V, Giannakas A, Deligiannakis Y, Konstantinou I (2015). Adsorption of phenol and methylene blue from aqueous solutions by pyrolytic tire char: Equilibrium and kinetic studies. Journal of Environmental Chemical Engineering.

[41] Mittal A, Mittal J, Malviya A, Gupta VK (2010). Removal and recovery of Chrysoidine Y from aqueous solutions by waste materials. Journal of Colloid and Interface Science.

[42] Gupta VK, Saleh TA (2013). Sorption of pollutants by porous carbon, carbon nanotubes and fullerene-An overview. Environmental science and pollution research.

[43] Gupta VK (2013). Adsorptive removal of dyes from aqueous solution onto carbon nanotubes: a review. Advances in Colloid and Interface Science.

[44] Saleh TA, Gupta VK (2012). Synthesis and characterization of alumina nano-particles polyamide membrane with enhanced flux rejection performance. Separation and purification technology.

[45] Gupta, V. K. & Saleh, T. A. Characterization of the bonding interaction between alumina and nanotube in MWCNT/alumina composite. *Current Nanoscience* **8**(5), 739–743 (2012).

