# Supplementary material for: Dimethyl diallyl ammonium chloride and diallylamin Co-polymer modified bio-film derived from palm dates for the adsorption of dyes
Source: Sci Rep. 2017 Oct 31;7:14448. doi: 10.1038/s41598-017-14327-7 (PMC5663925; doi:10.1038/s41598-017-14327-7)
Supplement: Supplementary file 1 — Supplementary file [file 41598_2017_14327_MOESM1_ESM.pdf]

Dimethyl diallyl ammonium chloride and diallylamin Co-polymer modified bio-film derived from palm dates for the adsorption of dyes

Mahjoub Jabli[2,3], Tawfik A. saleh[1*], Nouha Sebeia[2], Najeh Tka[3], Ramzi Khiari[4]

[1] Chemistry Department, King Fahd University of Petroleum & Minerals, Dhahran 31261, Saudi Arabia
[2]Textile Materials and Research, National School of Engineering (ENIM), Monastir 5000, Tunisia
[3] Laboratory of Organic Assymetric and Homogenous Catalysis (FSM), Monastir, Tunisia
[4] High Institute of Technological Studies of Ksar Hellal 4018, Monastir, Tunisia

**Corresponding author*

E-mail address: tawfik@kfupm.edu.sa ; tawfikas@hotmail.com

Kinetic modeling

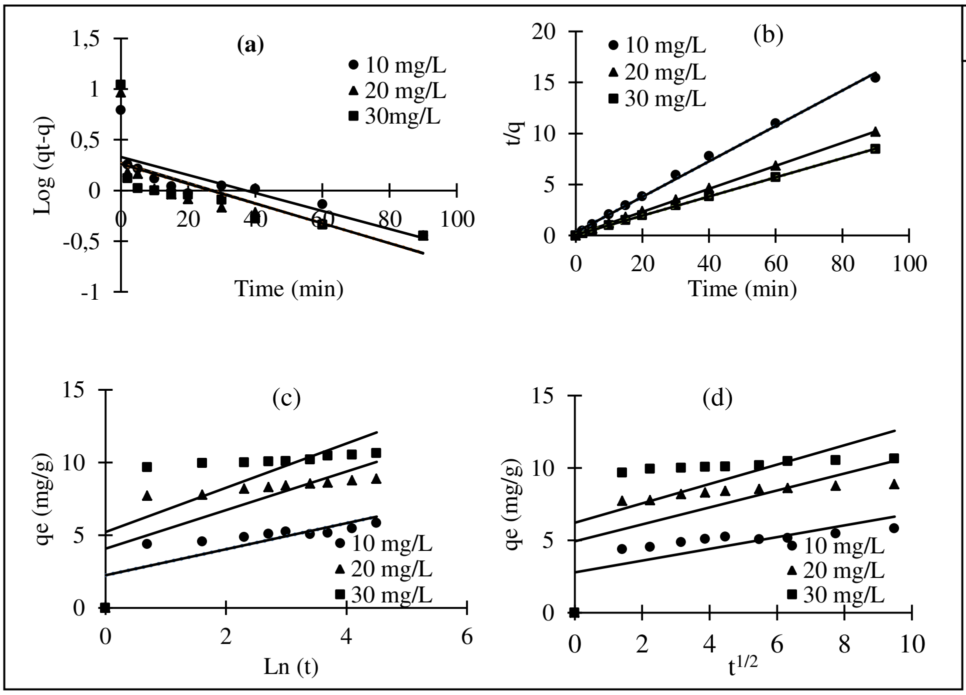

1

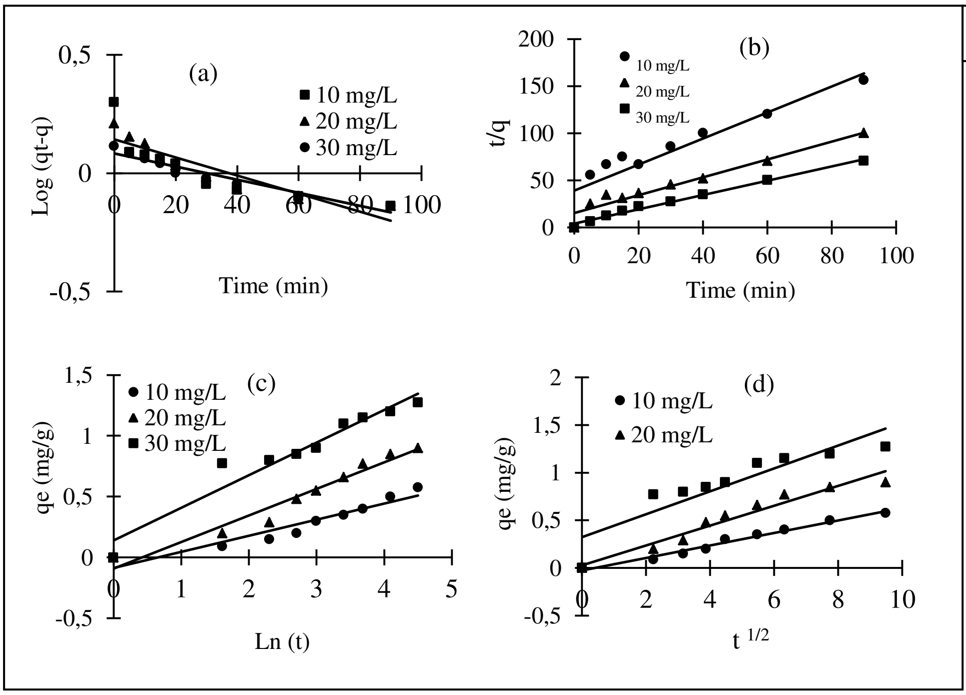

2

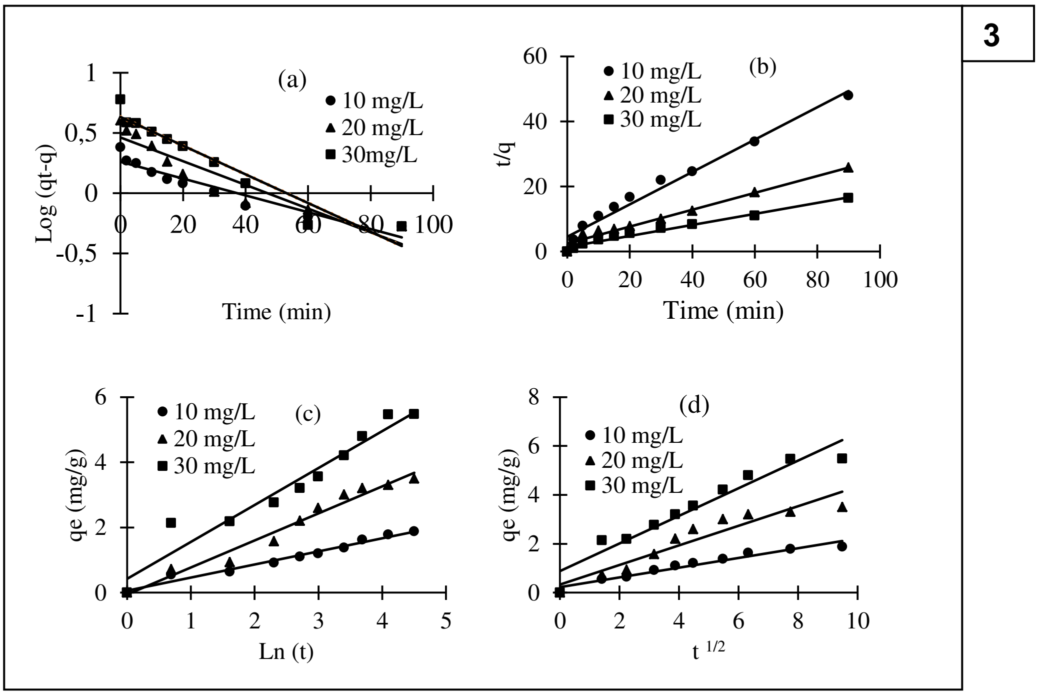

Figure S1. Linearized data through: (a) pseudo-first-order, (b) pseudo-second-order, (c) Elovich and (d) intra-particular diffusion for the adsorption of : **(1)** MB, **(2)** DY50 and **(3)** RB198 on unmodified film waste.

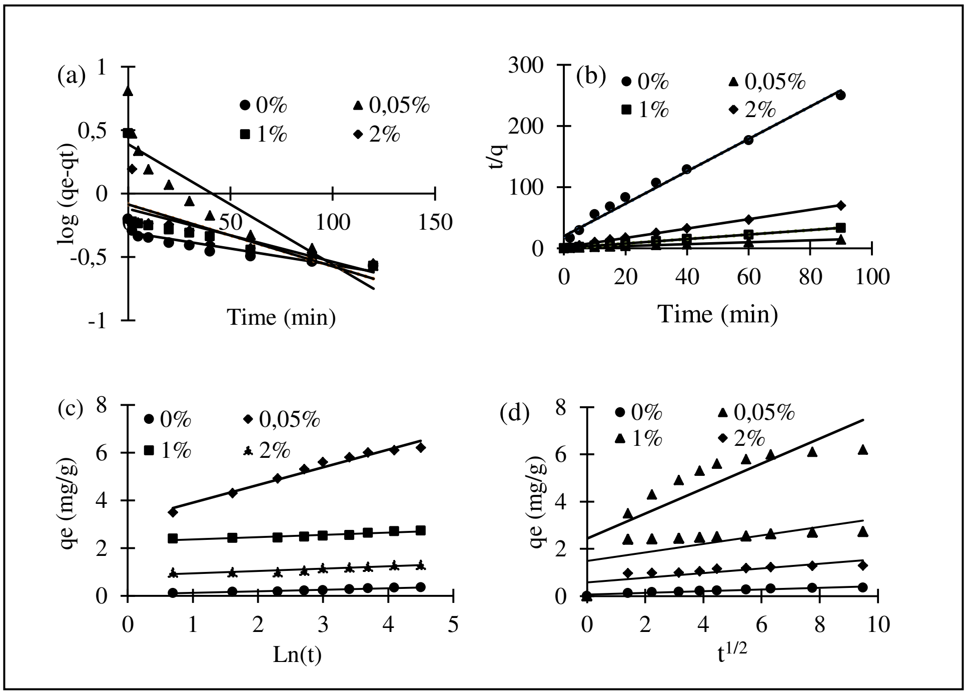

Figure S2. Linearized data through: (a) pseudo-first-order, (b) pseudo-second-order, (c) Elovich and (d) intra-particular diffusion for the adsorption of NBB on functionalized film waste.

Isotherms modeling

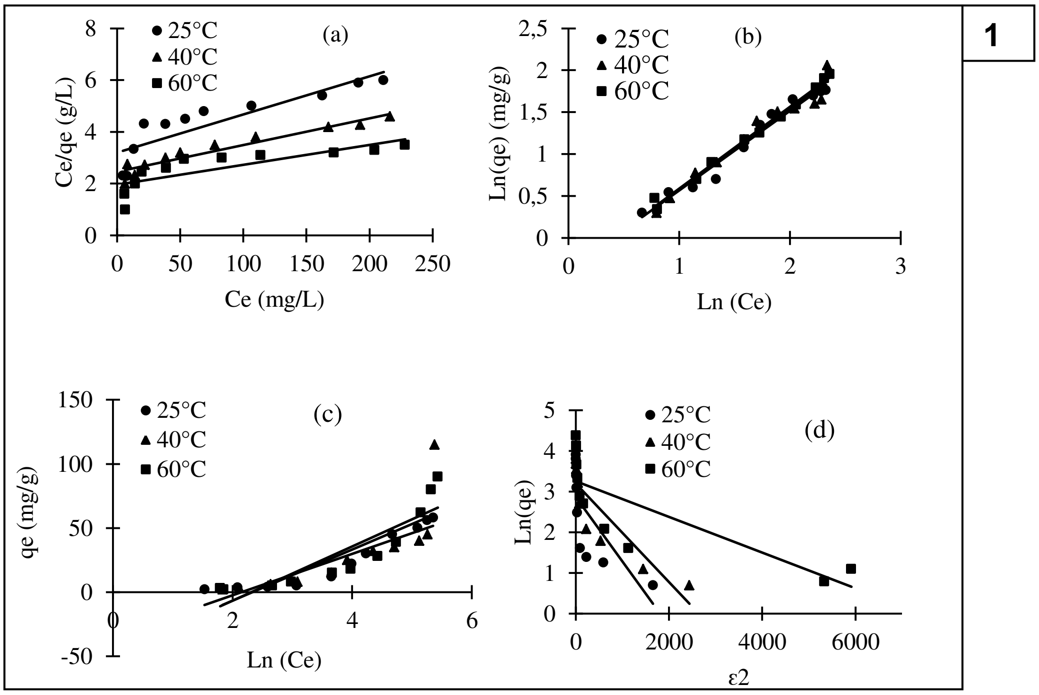

1

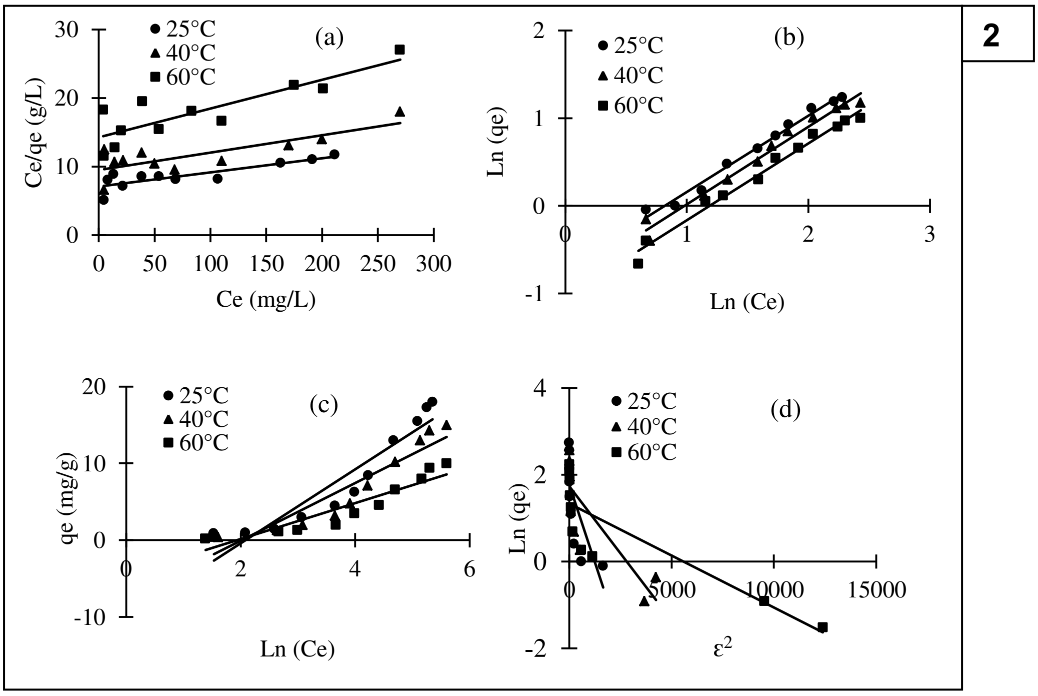

2

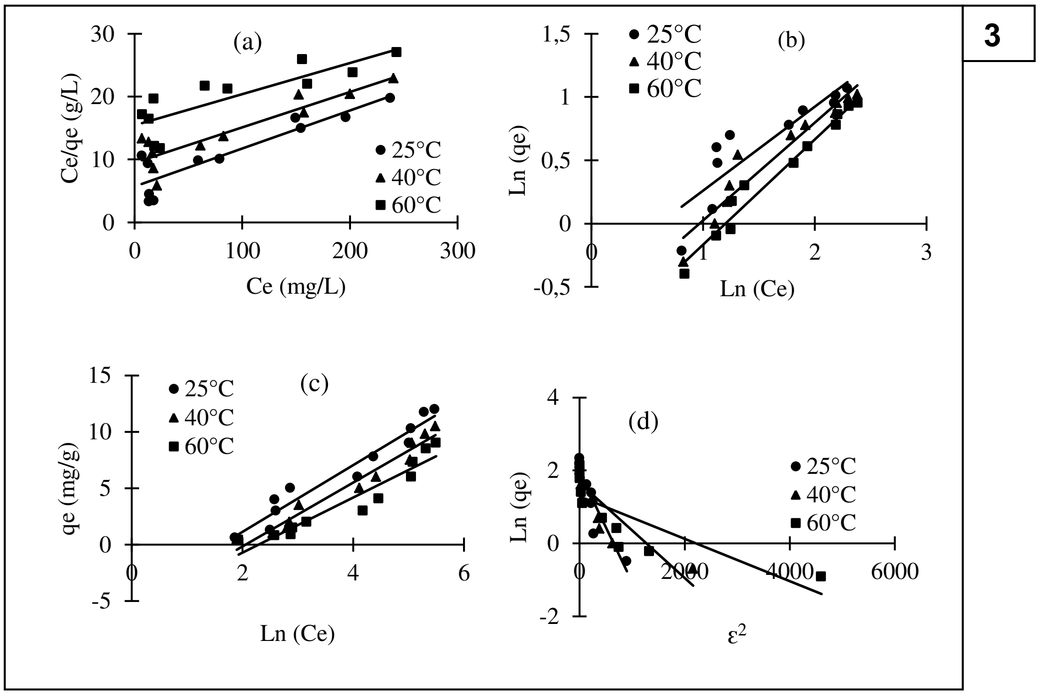

3

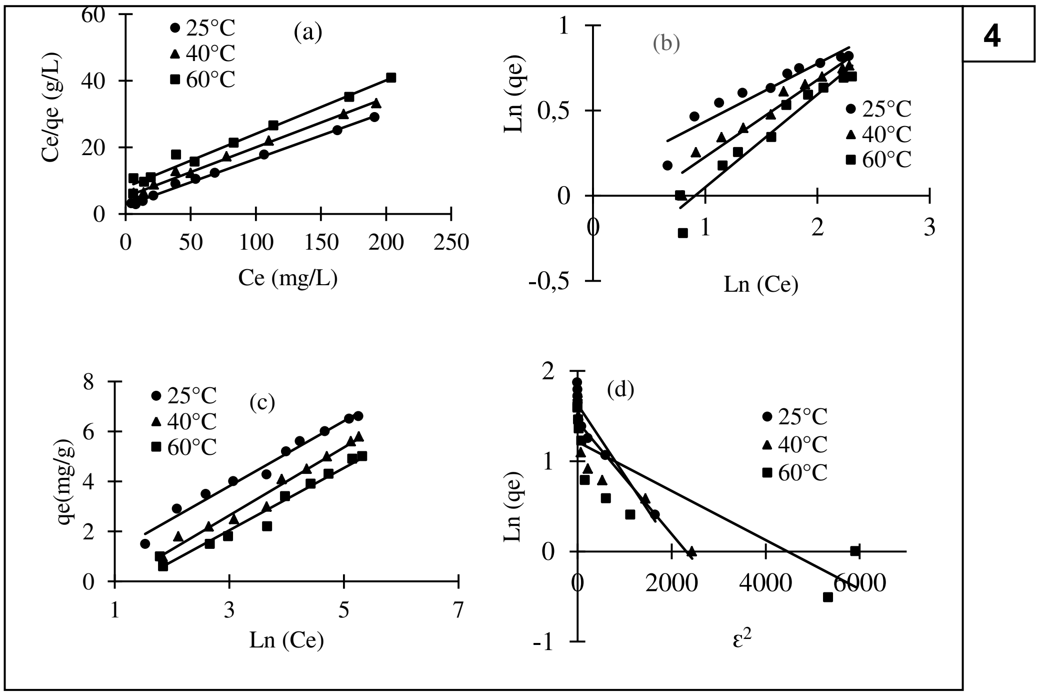

4

Figure S3. Linearized data for the adsorption of: **(1)** MB, **(2)** DY50, **(3)** RB198 and **(4)** NBB on unmodified film through: (a) Langmuir, (b) Freundlich, (c) Temkin and (d) Redushkevich equations.

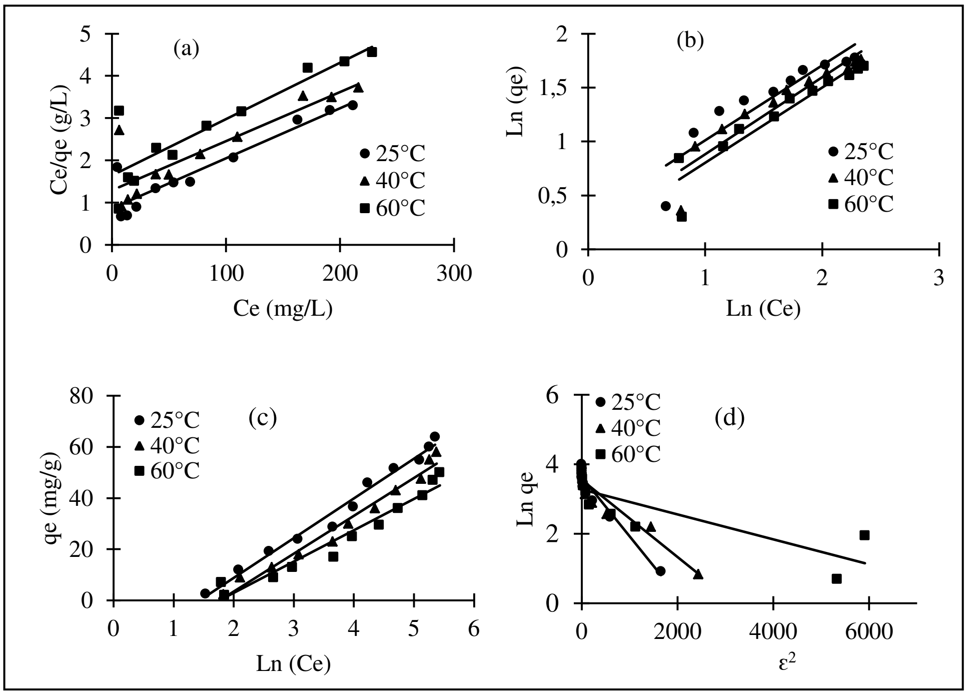

Figure S4. Linearized data for the adsorption of NBB on functionalized film (0.05%) through: (a) Langmuir, (b) Freundlich, (c) Temkin and (d) Redushkevich equations.

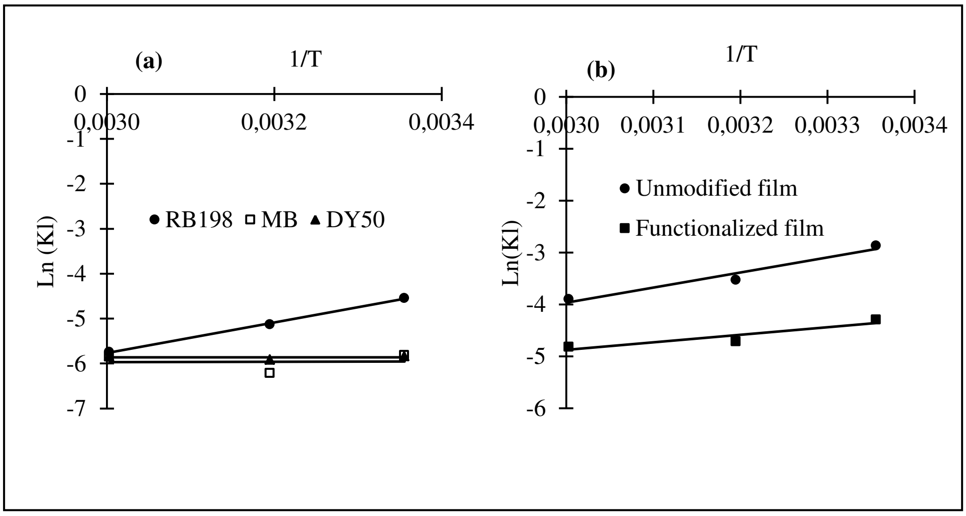

Figure S5. Plots of Ln (Kl) as a function of 1/T: (a) RB198, DY50, MB and (b) NBB.
